# Supplementary material for: Chalcone Scaffolds Exhibiting Acetylcholinesterase Enzyme Inhibition: Mechanistic and Computational Investigations
Source: Molecules. 2022 May 16;27(10):3181. doi: 10.3390/molecules27103181 (PMC9145706; doi:10.3390/molecules27103181)
Supplement: Supplementary file 1 [file molecules-27-03181-s001.zip › molecules-1694894-supplementary.pdf]

Supplementary Information

1) ¹HNMR and Mass Spectra of all the titled compounds (C1–C5)

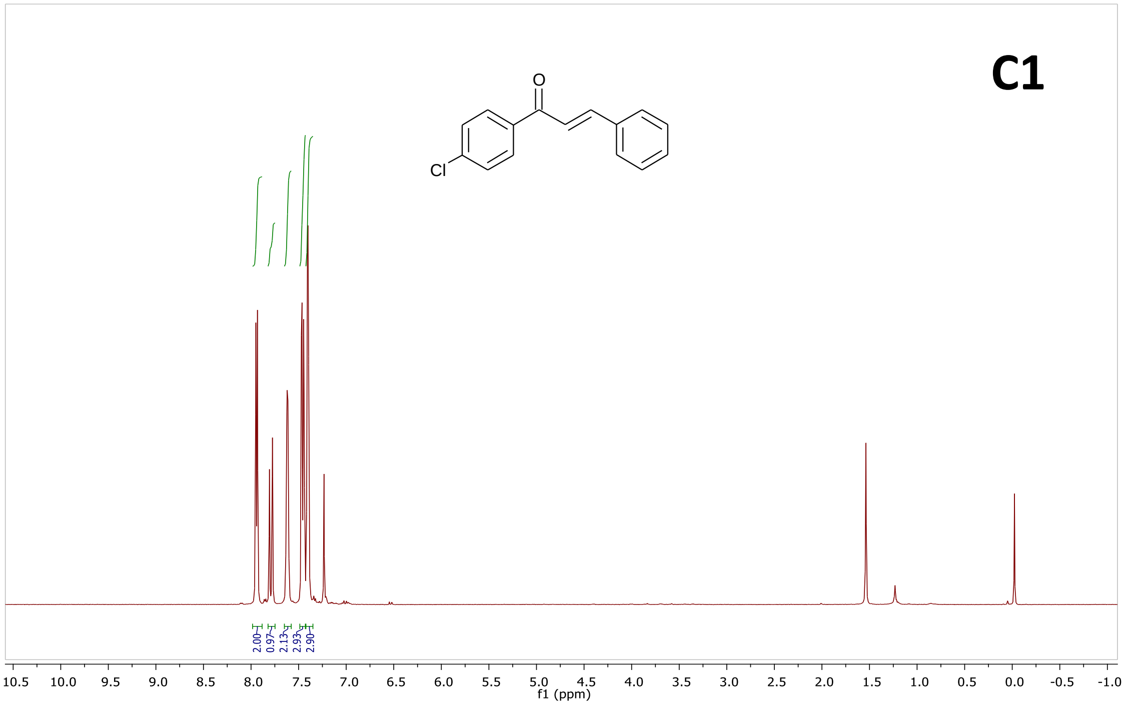

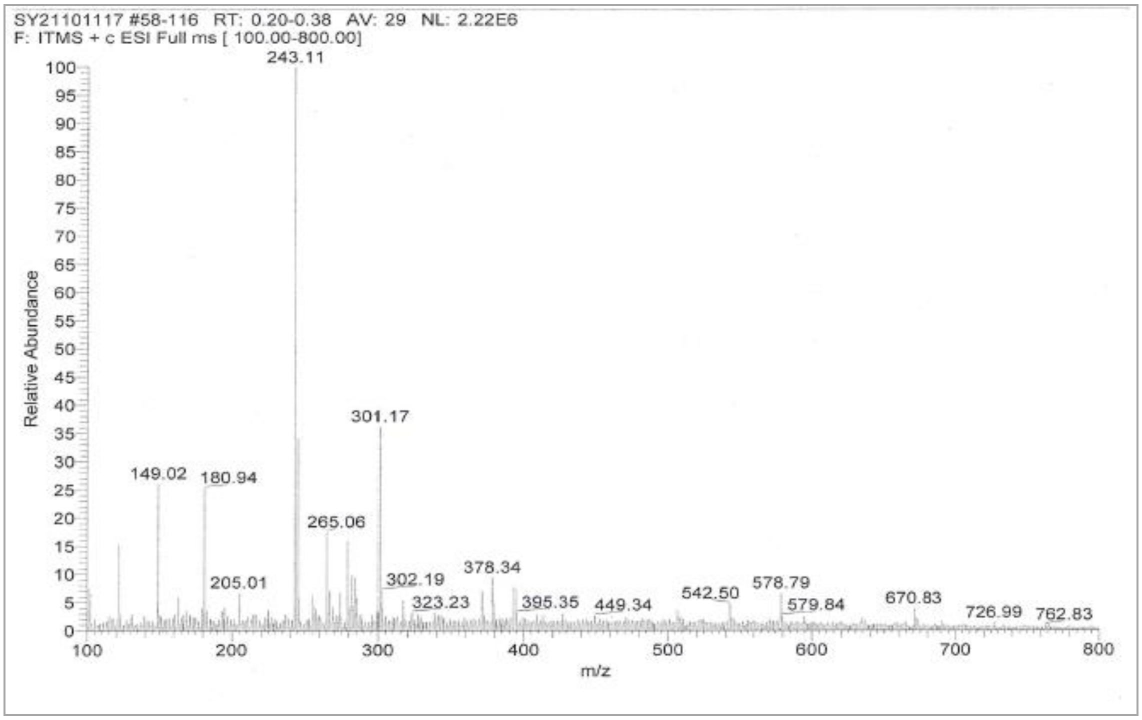

C2

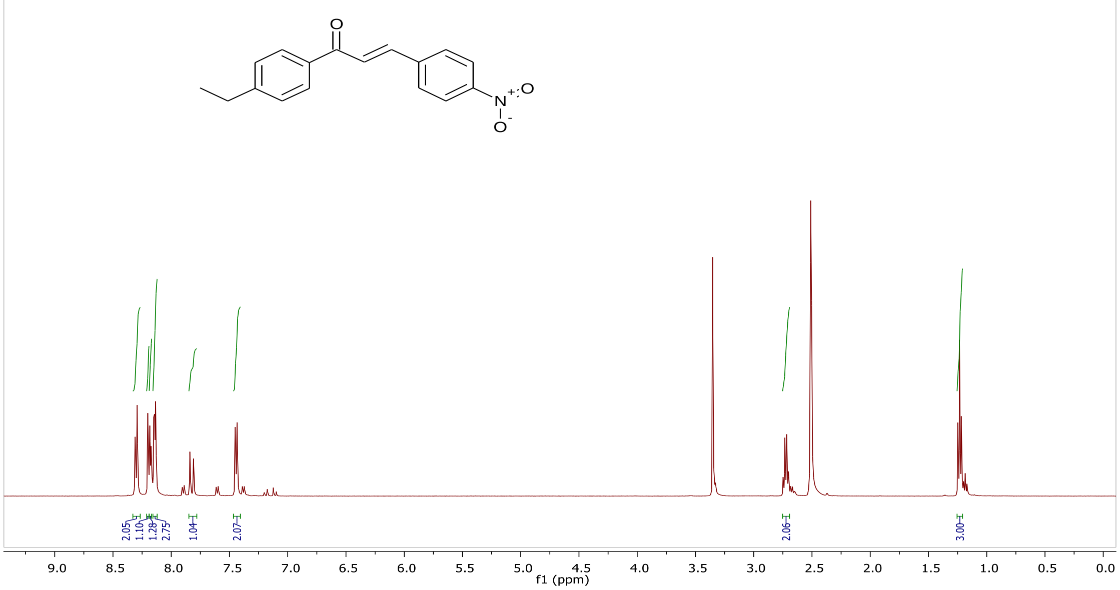
2.05
1.10
1.28
2.75
1.04
2.07
2.06
3.00
9.0
8.5
8.0
7.5
7.0
6.5
6.0
5.5
5.0
4.5
4.0
3.5
3.0
2.5
2.0
1.5
1.0
0.5
0.0
f1 (ppm)

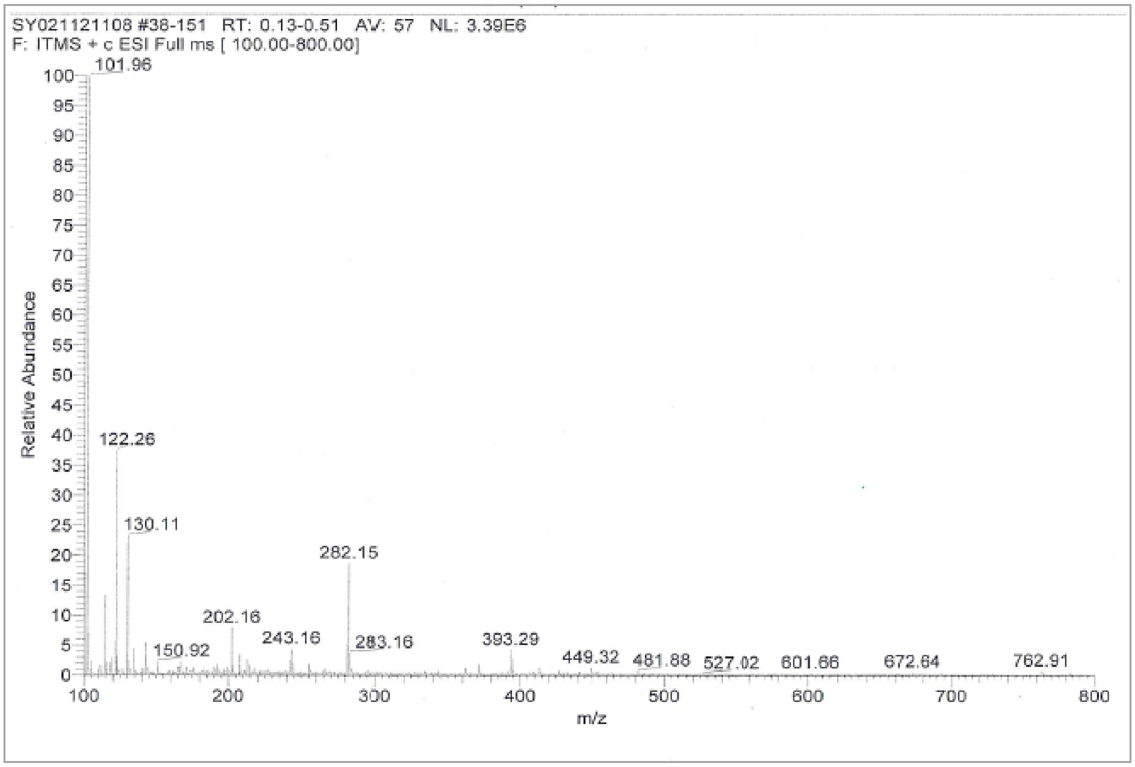
SY021121108 #38-151 RT: 0.13-0.51 AV: 57 NL: 3.39E6
F: ITMS + c ESI Full ms [100.00-800.00]
Relative Abundance
m/z
101.96
122.26
130.11
282.15
202.16
243.16
283.16
150.92
393.29
449.32
481.88
527.02
601.66
672.64
762.91

TAA-

C3

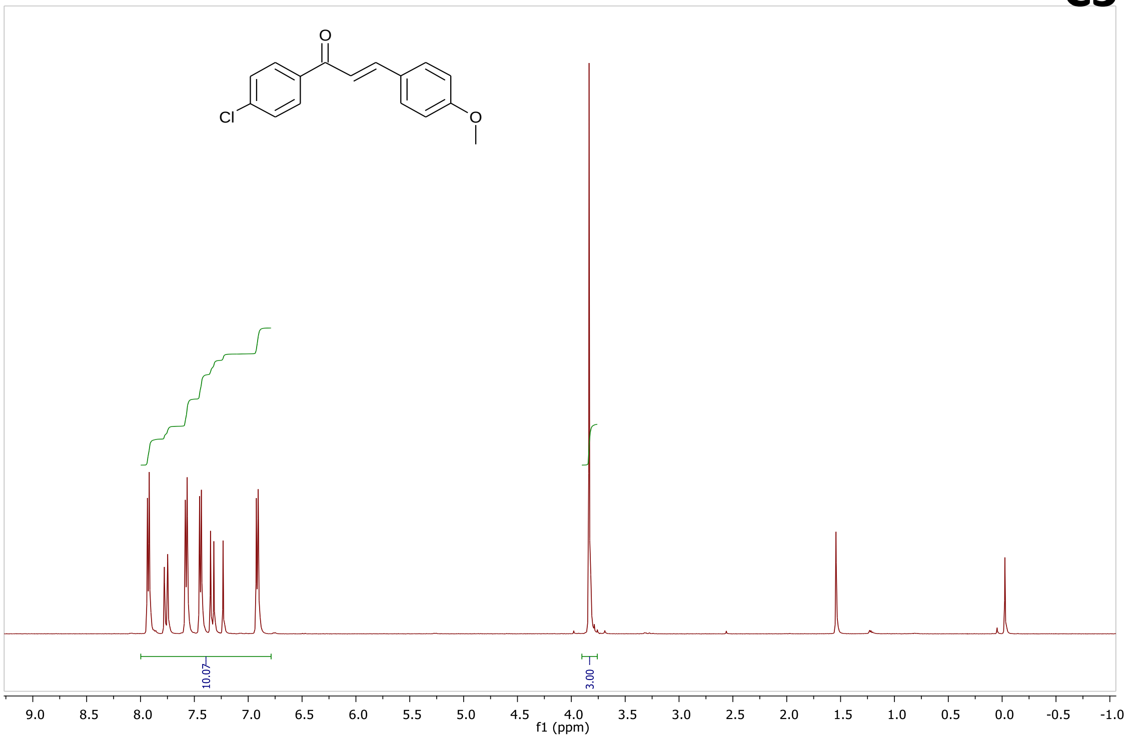
O
Cl
10.07
3.00
9.0
8.5
8.0
7.5
7.0
6.5
6.0
5.5
5.0
4.5
4.0
3.5
3.0
2.5
2.0
1.5
1.0
0.5
0.0
-0.5
-1.0
f1 (ppm)

003

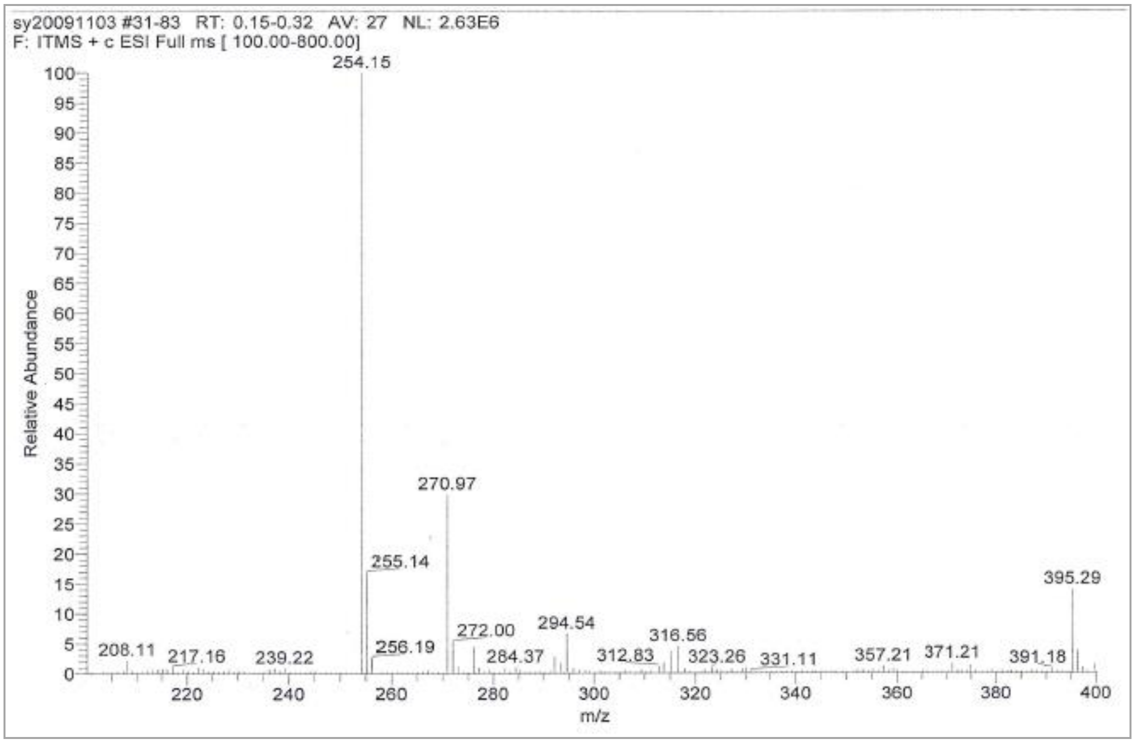
sy20091103 #31-83 RT: 0.15-0.32 AV: 27 NL: 2.63E6
F: ITMS + c ESI Full ms [100.00-800.00]
254.15
270.97
255.14
256.19
272.00
294.54
316.56
395.29
208.11
217.16
239.22
284.37
312.83
323.26
331.11
357.21
371.21
391.18
Relative Abundance
m/z

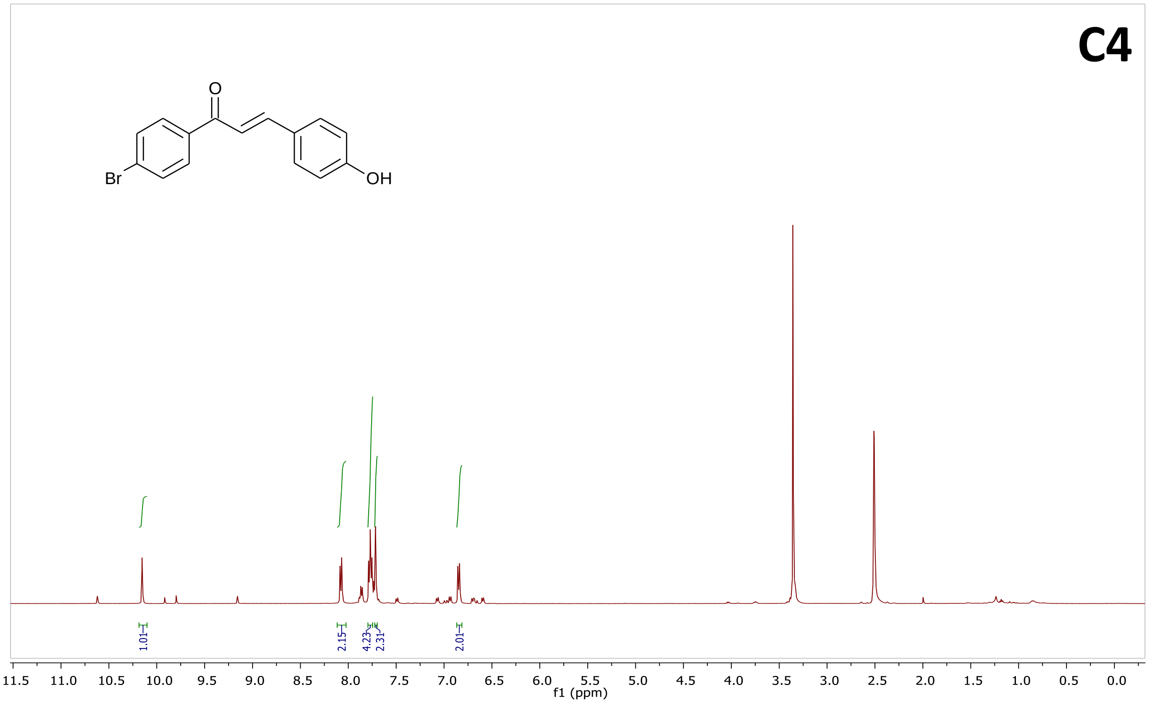

C4
O
Br
OH
1.01
2.15
4.23
2.31
2.01
11.5 11.0 10.5 10.0 9.5 9.0 8.5 8.0 7.5 7.0 6.5 6.0 5.5 5.0 4.5 4.0 3.5 3.0 2.5 2.0 1.5 1.0 0.5 0.0
f1 (ppm)

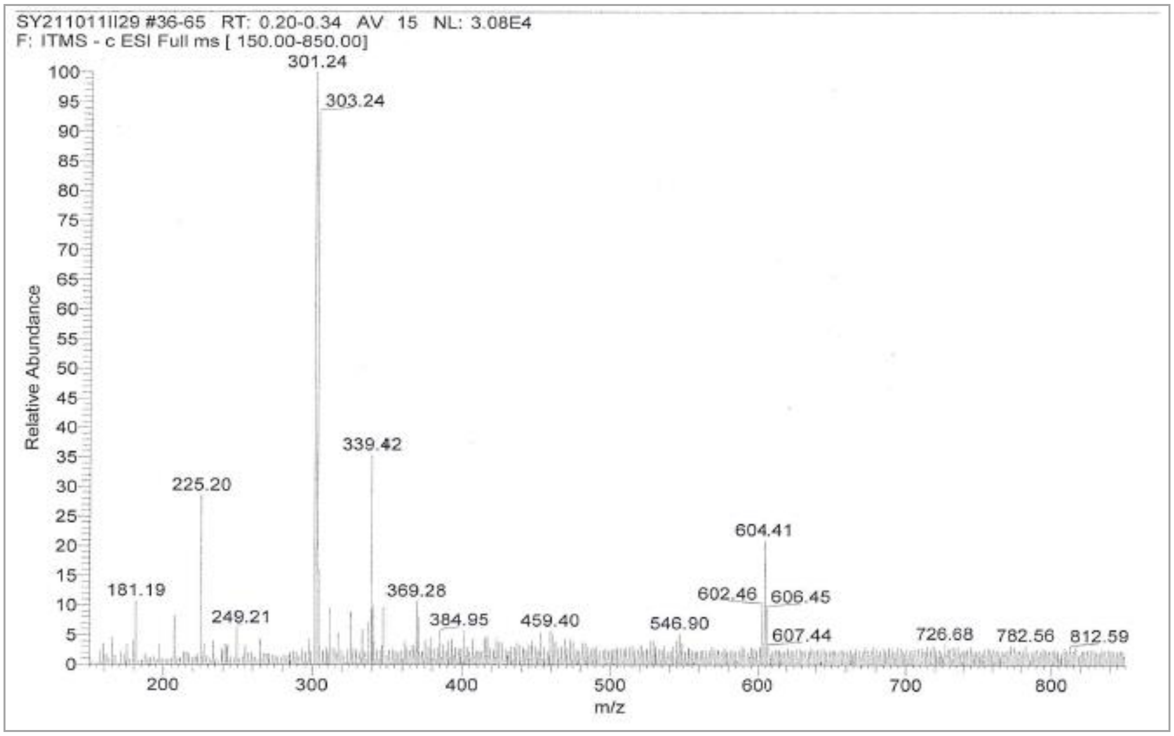

SY211011II29 #36-65 RT: 0.20-0.34 AV: 15 NL: 3.08E4
F: ITMS - c ESI Full ms [150.00-850.00]
Relative Abundance
301.24
303.24
339.42
225.20
604.41
181.19
369.28
602.46
606.45
249.21
384.95
459.40
546.90
607.44
726.68
782.56
812.59
m/z

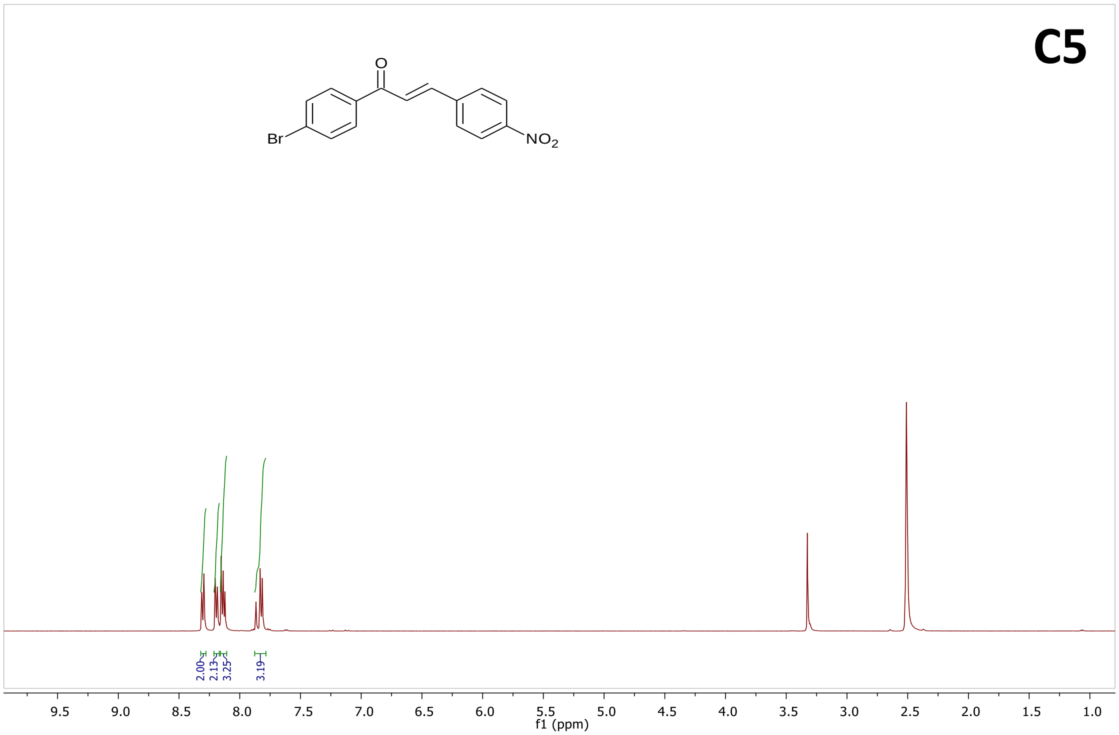

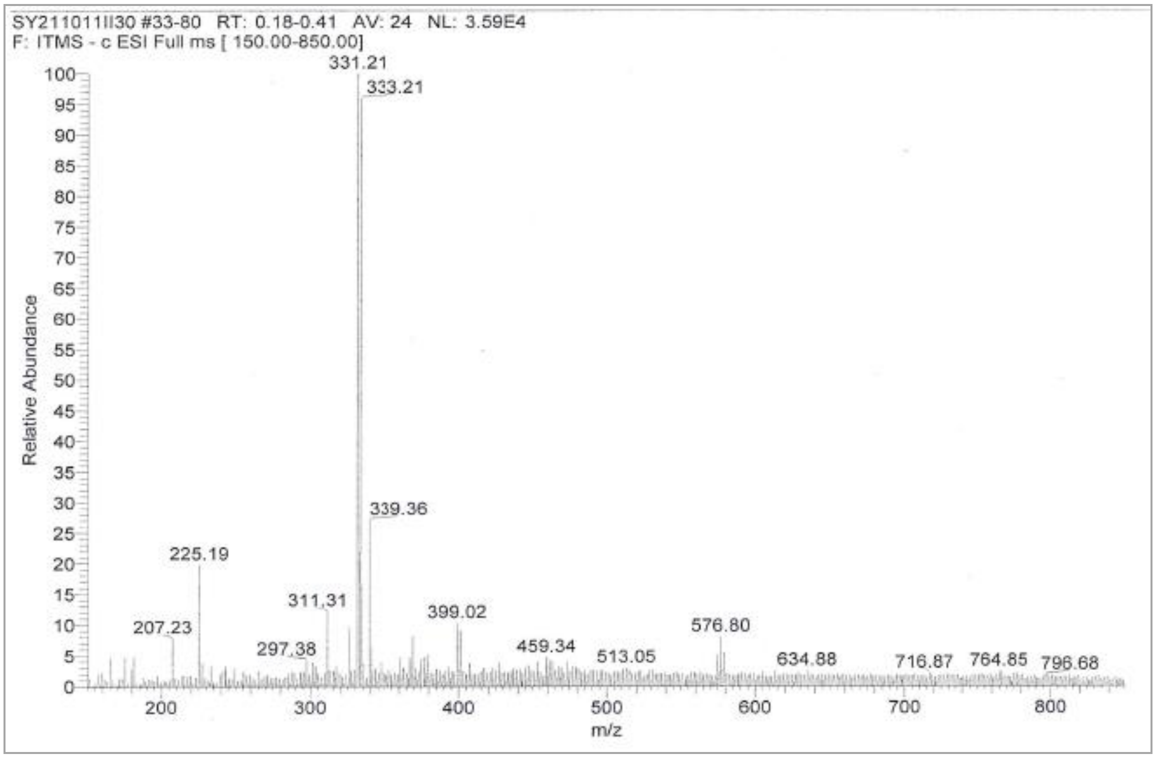

Figure S1. ^{1}HNMR and Mass Spectra of all the titled compounds (**C1–C5**).

2) MD simulations

Details about Periodic boundary conditions for apo form and complexes are as follow:

Periodic boundary condition for apo-form (AChE Enzyme**):**
cellBasisVector1 79.91699981689453 0 0
cellBasisVector2 0 78.48800277709961 0
cellBasisVector3 0 0 76.02799987792969
cellOrigin 8.65244197845459 69.42467498779297 60.14446258544922

Periodic Boundary conditions for MD simulations of complex (AChE Enzyme-**C4**)
Periodic Boundary conditions
cellBasisVector1 65.86899948120117 0 0
cellBasisVector2 0 62.15700149536133 0
cellBasisVector3 0 0 64.35200119018555
cellOrigin 4.822161674499512 65.49340057373047 56.73878479003906

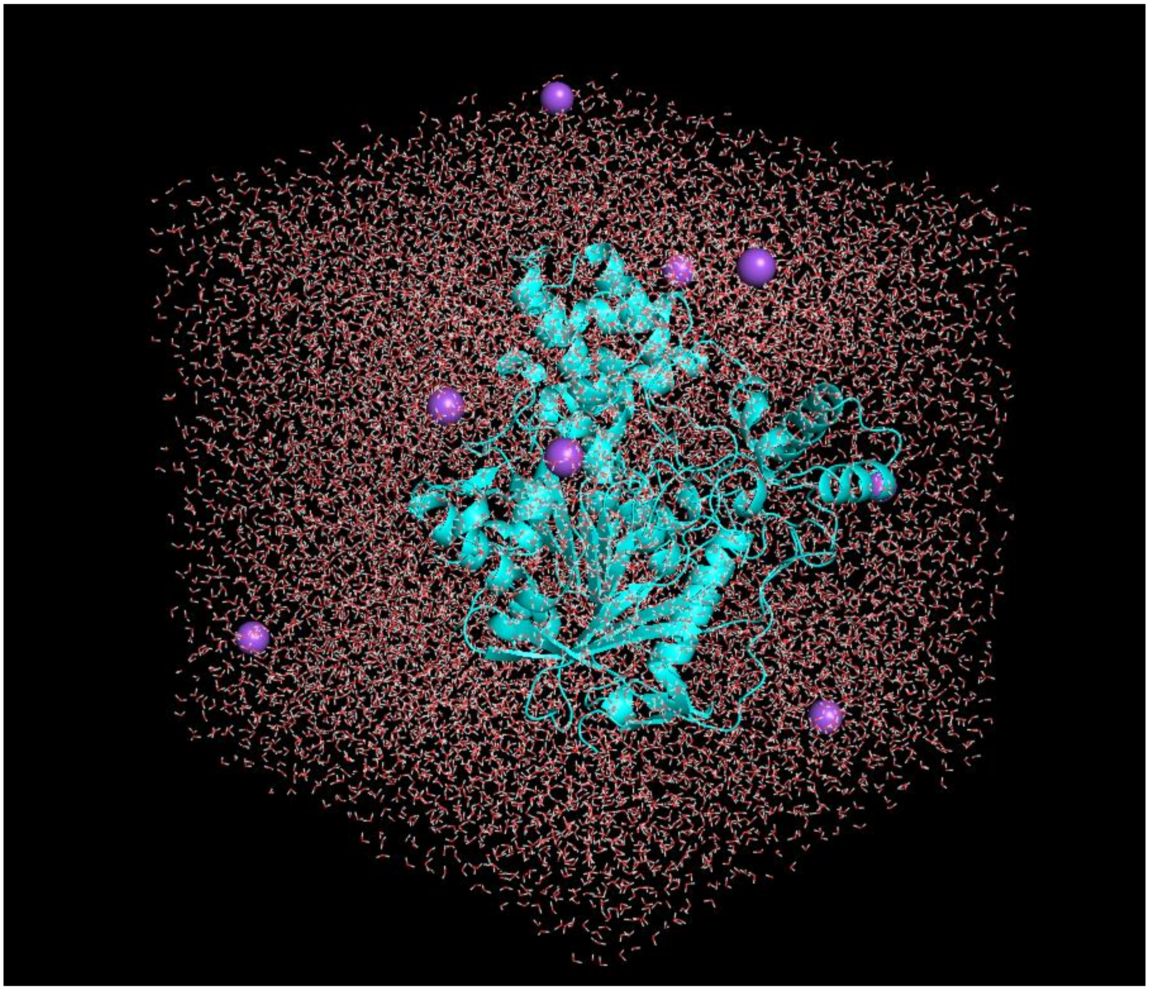

(a)

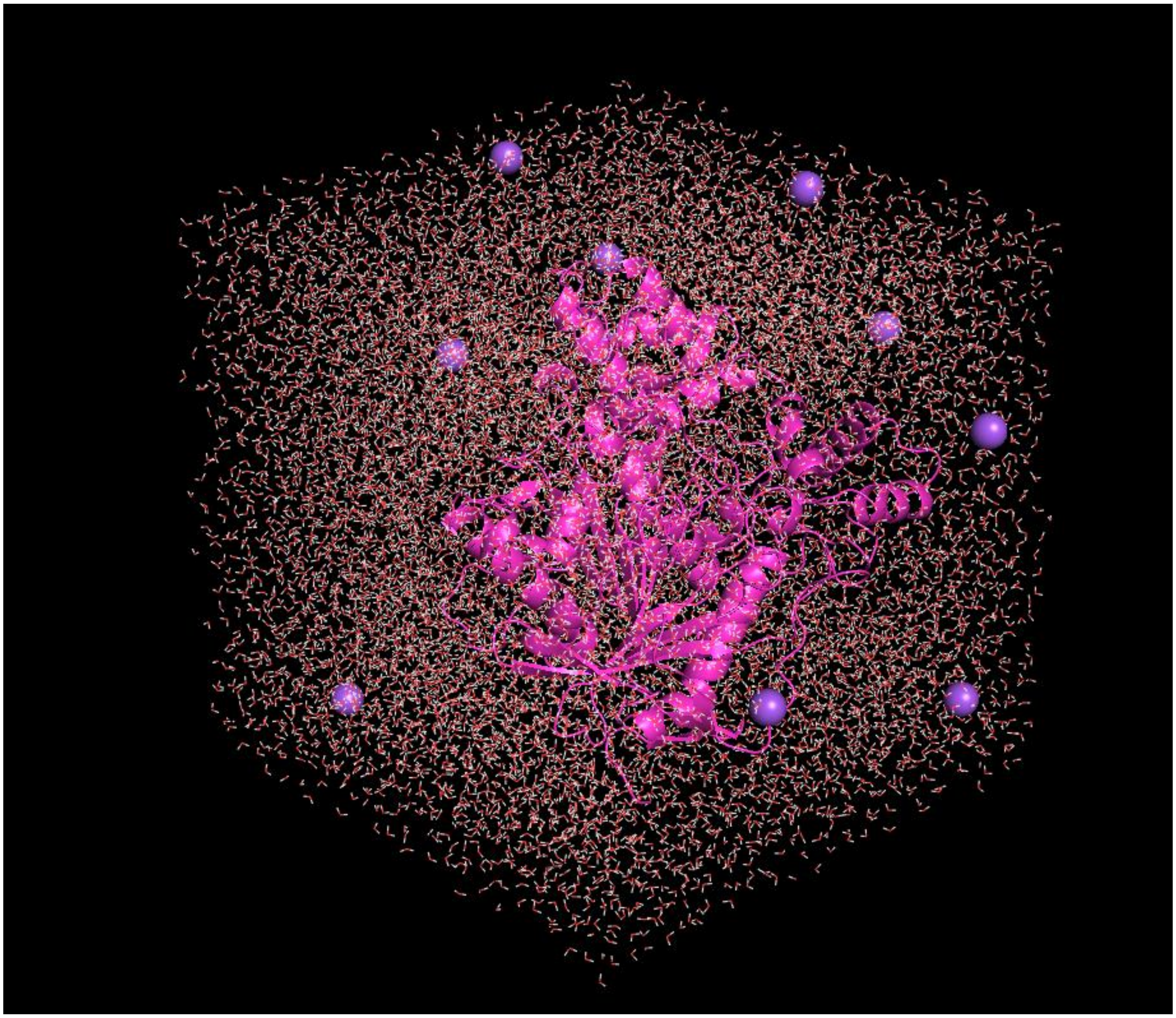

(b)

Figure S2. Solvated and ionized forms for (a) AChE Enzyme-**C4** complex and (b) AChE enzyme while performing MD simulations
